# Origin, Migration Routes and Worldwide Population Genetic Structure of the Wheat Yellow Rust Pathogen *Puccinia striiformis* f.sp. *tritici*

**DOI:** 10.1371/journal.ppat.1003903

**Published:** 2014-01-23

**Authors:** Sajid Ali, Pierre Gladieux, Marc Leconte, Angélique Gautier, Annemarie F. Justesen, Mogens S. Hovmøller, Jérôme Enjalbert, Claude de Vallavieille-Pope

**Affiliations:** 1 INRA UR 1290 BIOGER-CPP, Thiverval-Grignon, France; 2 Institute of Biotechnology and Genetic Engineering, the University of Agriculture, Peshawar, Pakistan; 3 Department of Agroecology, Aarhus University, Slagelse, Denmark; 4 UMR 8079 Ecologie Systematique Evolution, Univ. Paris-Sud., CNRS-F, Orsay, France; 5 Department of Plant and Microbial Biology, University of California, Berkeley, Berkeley, California, United States of America; 6 INRA UMR 320 Génétique Végétale, Ferme du Moulon, Gif sur Yvette, France; ETH Zurich, Switzerland

## Abstract

Analyses of large-scale population structure of pathogens enable the identification of migration patterns, diversity reservoirs or longevity of populations, the understanding of current evolutionary trajectories and the anticipation of future ones. This is particularly important for long-distance migrating fungal pathogens such as *Puccinia striiformis* f.sp. *tritici* (PST), capable of rapid spread to new regions and crop varieties. Although a range of recent PST invasions at continental scales are well documented, the worldwide population structure and the center of origin of the pathogen were still unknown. In this study, we used multilocus microsatellite genotyping to infer worldwide population structure of PST and the origin of new invasions based on 409 isolates representative of distribution of the fungus on six continents. Bayesian and multivariate clustering methods partitioned the set of multilocus genotypes into six distinct genetic groups associated with their geographical origin. Analyses of linkage disequilibrium and genotypic diversity indicated a strong regional heterogeneity in levels of recombination, with clear signatures of recombination in the Himalayan (Nepal and Pakistan) and near-Himalayan regions (China) and a predominant clonal population structure in other regions. The higher genotypic diversity, recombinant population structure and high sexual reproduction ability in the Himalayan and neighboring regions suggests this area as the putative center of origin of PST. We used clustering methods and approximate Bayesian computation (ABC) to compare different competing scenarios describing ancestral relationship among ancestral populations and more recently founded populations. Our analyses confirmed the Middle East-East Africa as the most likely source of newly spreading, high-temperature-adapted strains; Europe as the source of South American, North American and Australian populations; and Mediterranean-Central Asian populations as the origin of South African populations. Although most geographic populations are not markedly affected by recent dispersal events, this study emphasizes the influence of human activities on recent long-distance spread of the pathogen.

## Author Summary

Domestication of ecosystems, climate change and expanding global trade have accelerated the pace of disease emergence, caused by their introduction into new areas with susceptible hosts or the spread of new damaging pathogen genotypes. The wheat yellow rust pathogen (PST) is a pathogen with recent reports of invasions, significantly affecting worldwide wheat production. However, its origin and ancient migration routes remained unclear and the source areas of newly spreading strains were largely unknown. This information is important to understand the trajectories of current invasions and forecast the future spread, and more generally develop risk-assessment models of pathogen emergence. We analyzed a set of worldwide representative isolates of PST, which enabled to identify six different area-specific populations. Using population genetics tools, we identified its centre of diversity in the Himalayan and near Himalayan region. We also identified the source of the recently emerged populations; Middle East-East Africa as the source of high-temperature-adapted strains spreading worldwide; Europe as the source of American and Australian populations; and Mediterranean-Central Asian populations as the origin of South African populations. We demonstrate the influence of human activities on the recent long-distance spread of the disease, though most geographic populations are not markedly affected by recent dispersal events.

## Introduction

Domestication of ecosystems, climate change and the expanding global trade have accelerated the pace of pathogen emergence and spread [1], [2]. Widely distributed and genetically homogenous crop genotypes are conducive for rapid pathogen emergence and subsequent propagation across large areas. Even when novel pathogens are initially endemic to restricted geographical areas, they can rapidly emerge in new regions, provided they encounter a farmland with susceptible hosts and favorable environmental conditions. In widely grown food crops, many pathogens were introduced long ago, now being geographically widespread, and therefore they do not come to mind when considering invasive pathogens [3]. Although introductions could have occurred centuries ago, the evolution of such ubiquitous pathogens remains a significant cause of concern due to the risk of re-emergence caused by accidental dissemination of new, multi-virulent races [4], [5] or new, highly aggressive strains [6]. An accurate understanding of the origin, distribution of diversity reservoirs and past and recent migration routes of these pathogens is crucial to understand current epidemics, develop risk-assessment models and alleviate the potential adverse effects of disease emergence [7], [8]. This is particularly true for pathogens capable of long-distance migration, for which any newly advantageous mutant (increased virulence, aggressiveness or resistance to fungicides) has the potential to spread over large geographical area [9].

Yellow (stripe) rust on wheat, which is caused by *Puccinia striiformis* f.sp. *tritici* (PST), is present in most wheat-growing regions of the world [5], [10], [11], [12], [13], [14]. The pathogen has major negative impacts on wheat production due to re-emergences and invasions [5], [6], [15]. As for most ubiquitous pathogens of major crops, the origin, introduction pathways and current population structure of wheat yellow rust remains largely unknown. Although an origin in Transcaucasia has been hypothesized based on disease prevalence and geographical barriers [16], [17], it has never been assessed in light of new knowledge on the population structure of PST. Long-distance dispersal by wind is thought to play a key role in the dissemination of the disease. The fungus is capable of long-distance migration, with well-documented cases of recurrent re-establishment of pathogen populations in areas where there are no host plants during summer/winter to allow the pathogen survival, as for the main wheat-growing provinces of north-eastern China [9]. Such spread can be due to successive jumps from field to field by this polycyclic disease throughout the season (as in the USA [18]), as well as direct long-distance migration caused by winds, as documented between England and Denmark [19]. Accidental spore transport via human travel may also contribute to the intercontinental dispersal of the pathogen, as exemplified by the introduction of PST into Australia in 1979 from Europe through contaminated clothing or goods [15].

Despite the capacity for long-distance migration, the worldwide spread of PST is relatively recent, with most emergences reported only within the last decades. The pathogen reached South Africa in 1996 from an unknown source, but the first pathotypes detected were similar to those present in the Middle East and Mediterranean regions [6], [20]. PST was first reported in South America in the early 20^th^ century, with an unknown origin [16], [21]. More recently, an expansion of the geographic range of PST into the warm climate of south-eastern USA [22] was shown to be due to the emergence of an aggressive strain adapted to higher temperatures than usually reported to be optimal for PST [23], [24]. The same genotype was found in Australia two years later, while another closely related one was observed in Europe, Central and West Asia and East Africa [6]. In addition to recently colonized areas, the disease is known to periodically re-emerge through the acquisition of new virulences. These events are well documented for pathotypes carrying virulence against resistance gene *Yr9*, with a first report in 1986 in Eastern Africa (Ethiopia) and subsequent invasions of the Middle East, Pakistan and India, reaching Bangladesh in only 12 years [5]. The geographical origins of most of the emerging strains are unknown. The population structure of PST is therefore likely to display the hallmarks of a complex mixture of re-emergences over continuous wheat-growing areas and rare founder events due to long-distance migration. Recent spreads of the disease are likely to induce marked changes in patterns of population differentiation among regions, potentially erasing the signature of more ancient colonization events.

Several recent studies, using different genetic markers, investigated the population structure of PST over relatively large geographic scales. Analyses revealed an overall clonal population structure with low genetic diversity worldwide [15], [25], [26], [27], except China and Pakistan [28], [29]. Continental dispersal of the pathogen was well evidenced [30], leading to the conclusion that a newly emerged strain/pathotype can sweep all the worldwide populations [9], [26]. However, despite the importance of this pathogen and increased research efforts, aspects of the worldwide population structure of the pathogen, both in terms of population subdivision and diversity, remain undetermined.

In the present study, we assembled a representative set of isolates from a larger collection of PST populations from the worldwide geographical range of the fungus and analyzed their genetic variability using a single set of highly variable genetic markers. Our objectives were: (i) to identify the main genetic groups in modern PST populations; (ii) to test the Himalayan region as putative centre of diversity; and (iii) to identify the geographical origin of recently emerged populations and investigate the ancestral relationships among geographically spaced populations.

## Materials and Methods

### Selection of isolates

A set of 409 isolates was selected to represent 11 geographical regions on six continents (Africa, Asia, Australia, Europe, North America and South America) from a collection of more than 4,000 isolates available at Institut National de la Recherche Agronomique (INRA), France and the Global Rust Reference Centre, Aarhus University, Denmark. The selection was made to maximize the representation of each population (partially assessed previously by AFLP, microsatellites and/or virulence profiles [6], [10], [25], [28], [29], [31], [32]), while balancing the number of isolates for each previously described genetic group, and trying to cover the geographic distribution of each. Isolates representative of aggressive strains were selected from the two recently emerged aggressive strains, PstS1 (associated with the post-2000 epidemics in the USA and Australia), and the Euro-Asian strain, PstS2, as well as a set of aggressive isolates frequently reported in Southern Europe, PstS3, which were less aggressive than PstS1 and PstS2 [24]. Isolates from the recently invading populations (e.g. South Africa) were included to infer on their source using non-parametric and Structure analyses, and were therefore not included in population-based analyses. Details regarding the number of isolates are shown in Table 1.

**Table 1:** Sampling regions and number of isolates selected for the analysis of the worldwide population structure of wheat yellow rust pathogen *Puccinia striiformis* f.sp. *tritici*.

Geographical origin	Country	Number of isolates	Sampling year
**Older native populations**			
**South-East Asia**	China	71	2004–05
	Nepal	55	2008
	Pakistan	68	2004–2006, 2008
**Middle-East**	Afghanistan	7	2009
	Cyprus	9	2005–2006
	Iran	17	2005
	Israel	7	2005–2006
	Lebanon	5	2006
	Turkey	10	2005
	Yemen	12	2003, 2005
**East Africa**	Eritrea	23	2002–2005
**Central Asia**	Azerbaijan	11	2005
	Kazakhstan	6	2005
	Kyrgyzstan	7	2005
	Uzbekistan	1	2003
**Mediterranean area**	Algeria	7	2005–2006
	Italy	2	1998–2006
	Morocco	2	2006
	Portugal	4	2006
	Spain	2	2006
	Tunisia	4	2005, 2007
**NW Europe**	Denmark	7	1995–2002
	France	5	1997–2008
	United Kingdom	5	1975, 1978, 1991–1998
**Recent invasions**			
**North America**	Mexico	2	1989, 2002
	USA	10	1981–1983, 1991–1997
**South America**	Argentina	1	2010
	Brazil	6	2010
	Chile	7	2010
	Uruguay	10	2010
**South Africa**	South Africa	6	1996–2004
**Aggressive isolates**	Australia	4	2002–2004
	Denmark	7	2001–2006
	France	1	1997
	Mexico	2	2002–2003
	USA	6	2000–2005
**Total**		409	

### Molecular genotyping

For most isolates, DNA was already available, having been previously extracted through modified CTAB protocols [19], [33]. For isolates from Pakistan (2008), Nepal (2008) and China (2005), DNA was extracted from 5 mg of spores following Ali et al. [34]. All of the isolates were multiplied from single pustule lesions to avoid a mixture of genotypes. Molecular genotyping was carried out using a set of 20 microsatellite loci in three multiplex reactions, with subsequent separation of the PCR products using a Beckman Coulter CEQ-8000 DNA Analyzer. Electrophorograms were processed using the CEQ-8000 Genetic Analysis System Software (Beckman Coulter [34]).

### Analyses of population subdivision

We investigated the existence of different genetic pools of PST using both multivariate and model-based Bayesian clustering approaches. These methods avoid the clustering of individuals on a priori knowledge such as geographical locations that may artificially group different genetic lineages introduced in the same area, hindering the detection of admixture events among them [35]. Multivariate analyses were carried out using discriminant analyses of principal components, implemented in the Adegenet package in R environment [36]. The number of clusters was identified based on the Bayesian Information Criterion (BIC), as suggested by Jombart et al. [36].

The model-based Bayesian method implemented in Structure 2.2 [37] was mostly used to confirm results of multivariate analyses, bearing in mind that this method makes the assumption of linkage equilibrium and that violations of this hypothesis for instance due to asexual reproduction can lead to spurious assignments and overestimate the number of clusters [38]. The rationale of this method is to assign multilocus genotypes to different clusters while minimizing the Hardy-Weinberg disequilibrium and the gametic phase disequilibrium between loci within clusters (where the number of clusters may be unknown). The Monte Carlo Markov Chain (MCMC) sampling scheme was run for 200,000 iterations with a 100,000 burn-in period, with K ranging from 1 to 10 and 20 independent replications for each K. The Structure outputs were processed with Clumpp [39]; a G′-statistic greater than 80% was used to assign groups of runs to a common clustering pattern.

The relatedness among geographically spaced populations was plotted using a neighbor-joining population tree based on the genetic distance *D_A_* [40], as implemented in the Population program [41]. Significance was assessed using 1,000 bootstraps. The level of population differentiation was assessed using pairwise *F_ST_* statistics among pairs of populations (GENETIX 4.05.2 [42]).

### Analyses of genetic variability and recombination

The quality of the set of markers, with respect to the inference of population structure, was tested by assessing the ability of the set of microsatellite loci to detect multilocus genotypes (MLGs) under panmixia, using Genclone [43]. The redundancy of the set of loci was tested by estimating the linkage disequilibrium among different loci and generating 1,000 random permutations with Genetix 4.05.2 [42]. Within-population variability was assessed using allele richness and gene diversity, calculated with Fstat 2.9.3 [44]. Genotypic diversity was estimated with MultiLocus 1.3 [45]. Private allelic richness was estimated using a rarefaction approach, implemented in Adze [46]. Observed (Ho) and unbiased expected heterozygosity (He) were computed using Genetix 4.05.2 [42]. The null hypothesis of the Hardy-Weinberg equilibrium within each population was tested using the exact test implemented in Genepop 4.0 [47]. Calculations were performed both on the whole dataset and on the clone-corrected data (i.e., a dataset in which only one representative of each repeated MLG is kept). Only the clone-corrected data are reported in cases where the two datasets yielded different results because the sampling during epidemics would result in over-representation of certain clones due to the recent clonal burst at local and seasonal scale, which may bias the population genetic analyses [48].

### Ancestral relationship and migration patterns among populations

We used approximate Bayesian computation (ABC) to compare different competing scenarios describing ancestral relationship among populations. The approach bypasses the calculation of exact likelihoods rendering it efficient for complex population genetic models such as those underlying biological invasions [49], [50]. The rationale is to simulate datasets assuming different parameter values under different scenarios to estimate posterior probabilities of competing scenarios and the posterior distributions of the demographic parameters under a given scenario using comparisons between simulated and observed data sets based on summary statistics (e.g. genetic distance between populations). As different scenarios compared were defined based on the results of population structure analyses, they are therefore described in the results section. These scenarios globally contrasted various hypotheses concerning the source and sink relationships among the different genetic groups identified using clustering methods.

Simulations were performed using Diyabc [51], model selection and parameter estimation was carried out using the abc package in R [52]. A total of 5×10^5^ simulated data sets was generated for each scenario under the generalized stepwise mutation model, with two parameters: the mean mutation rate (*I*) and the mean parameter (*P*) of the geometric distribution used to model the length of mutation events (in number of repeats). The mean mutation rate was drawn from a uniform distribution of 10^−4^ to 10^−3^, while the mutation rate of each locus was drawn from a gamma distribution (mean = *μ*, shape = 2). The parameter *P* was kept in the range of 0.1 to 0.3. A range of 40 contiguous allelic states was kept for each locus, characterized by individual value of mutation rate (*l_L_*) and the parameter of the geometric distribution (*P_L_*), which were obtained from Gamma distribution (with mean = 1, range 5×10^−5^ to 5×10^−2^ for *I_L_*; and mean = *P*, shape = 2, shape 0.01–0.09 for *P_L_*). Mean number of alleles per locus, mean genetic diversity [53], mean variance in allele size, genetic differentiation between pairwise groups, *F_ST_* [54] , and genetic distance (*δμ^2^* [55] were used as summary statistics.

Relative posterior probabilities of different scenarios were estimated by fitting a multinomial logistic regression between the summary statistics and a polychotomous variable corresponding to the different model indexes [56], using 1% of simulated dataset closest to the observed data. The posterior distributions of parameters were estimated for the most likely scenario using a local linear regression [57], [58] on 1% simulated datasets closest to the observed data. Confidence in model choice was assessed using a leave-one-out method [52] for each model we drew 100 of the 5×10^5^ simulated datasets used for model selection and treated them as observed datasets (i.e., pseudo-observed datasets). Posterior probabilities of competing models were evaluated for each pseudo-observed dataset, using all remaining simulated datasets and the same methodology as described for the observed dataset. Confidence in model choice was then estimated using the number of pseudo-observed dataset that gave higher posterior probability to the model they had been simulated with. In tests of goodness-of-fit (i.e., model checking), we simulated 100 datasets of similar number of markers as observed datasets and calculated for each dataset the average across loci of several test quantities. The set of test quantities included the summary statistics used in analyses of the observed dataset. However, because using the same statistics in parameter inference and model checking can overestimate the quality of the fit [51], we selected additional summary statistics that had not been used in parameter inferences: mean allele size variance across loci, mean index of classification, and for each population pair the mean gene diversity, mean allelic richness, and mean allele size variance across loci. Test statistics computed from observed data were then ranked against the distributions obtained from simulated datasets [58].

## Results

### Summary of genetic variation

We performed multilocus genotyping of 409 PST isolates, representatives of a worldwide collection, using a set of 20 microsatellite markers. Plotting the multilocus genotypes detected against the number of loci re-sampled showed that the full set of SSRs was sufficient to discriminate clonal lineages (supporting information; Fig. S1). No significant linkage disequilibrium was found among SSR loci (data not shown), suggesting a lack of redundancy among markers. Some of the loci were monomorphic in certain geographical areas, except that China had no such locus and Pakistan had only one (RJN-12; supporting information; Table S1).

### Population subdivision

**Figure 1:**
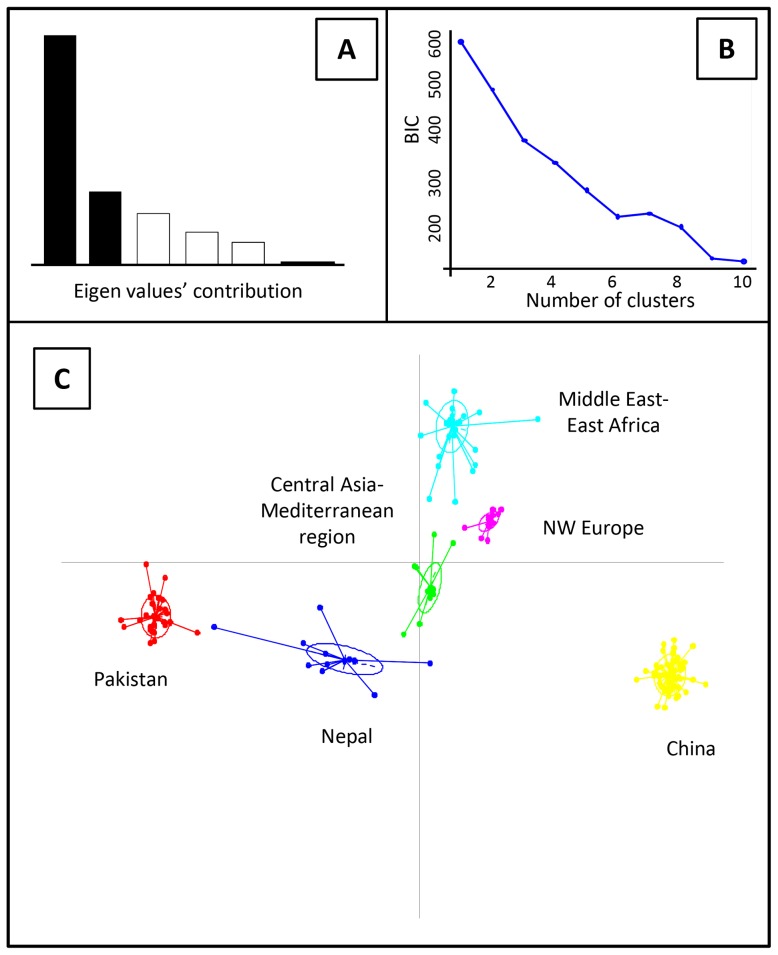
Discriminant analysis of principal components (DAPC) analysis of worldwide PST populations sampled from different geographical regions. The Eigen values of the analysis suggest that the first two components explained the maximum genetic structure of the dataset (A). The Bayesian information criteria (BIC) supported six distinct genetic groups (B). Scatter-plot of the worldwide distribution of PST isolates into six genetic groups (C).

**Figure 2:**
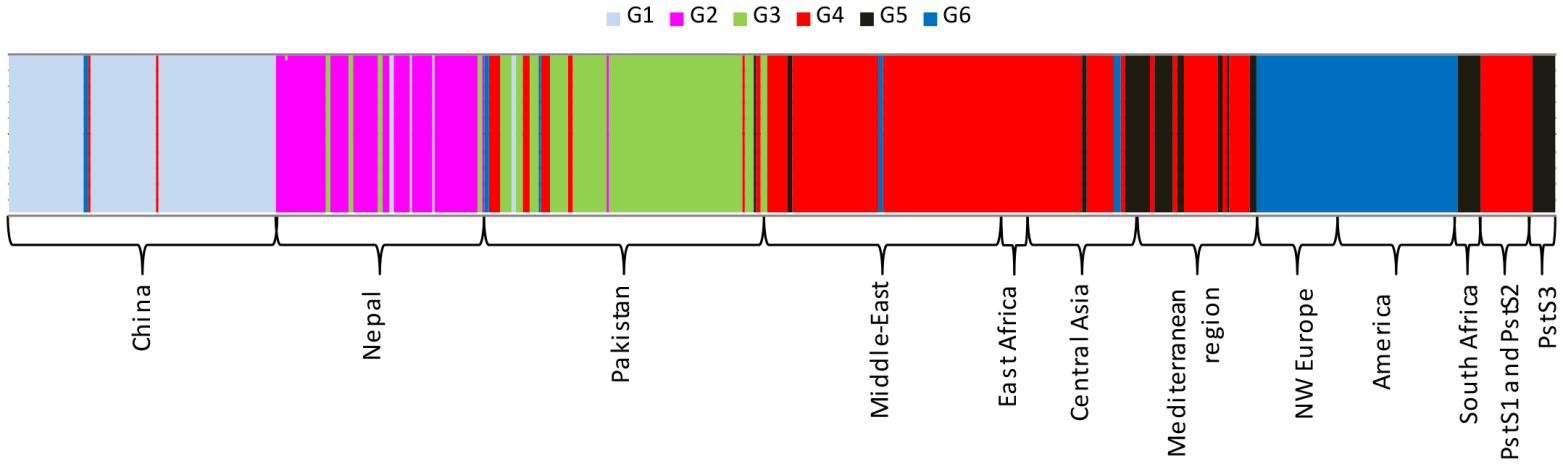
Clustering of 409 PST isolates representing worldwide geographical regions to genetic groups for the optimal K-value (K = 6) in the DAPC analysis. PstS1 and PstS2 refers to the two closely related aggressive strains, while PstS3 refers to the older aggressive isolates regularly reported in Southern Europe.

Genotypes clearly clustered according to their geographical origin in the non-parametric discriminant analysis of principal components (DAPC) analysis. The Bayesian information criteria (BIC) curve in the DAPC analyses supported *K* = 6 with a clear discrimination of genotypes from China, Pakistan, Nepal, Middle East-East Africa, North western (NW) Europe and Central Asia-Mediterranean region (Fig. 1 and Fig. 2).

Analyses using the model-based clustering method implemented in Structure also identified an optimal number of clusters (*K*) equal to 6, based on the rate of change in the log probability of data across successive *K* values [59], and patterns of subdivision were largely consistent with the results of non-parametric DAPC (Fig. 2 and Fig. S2). At *K* = 2, Middle Eastern, Mediterranean and Central Asian populations were assigned to one group; the Chinese population was assigned to the other group; and Nepalese, Pakistani and NW European populations had a mixed assignment of the two groups (Fig. S2). Increasing *K* to 3 individualized a Pakistan-specific group, while increasing *K* to 4 split the cluster of Middle East, Central Asia and Mediterranean region into two groups, one specific to the Middle East and East Africa and the other specific to the Central-Asia and Mediterranean region, with substantial admixture from the Middle East. The Middle Eastern and East African populations had no differentiation from each other and are termed as Middle East-East Africa, onward. At *K* = 5, the NW European populations were separated from the Chinese population, and at *K* = 6, the Nepalese group individualized (Fig. S2). Increasing *K* above 6 did not reveal any further subdivisions (Fig. S2).

Population differentiation among the different geographically spaced populations was estimated by means of pairwise *F_st_*. Populations showed a strong differentiation, with high and significant *F_st_* values for all pairs except for PST from the Middle Eastern, Central Asian and Mediterranean regions (Table 2), indicative of a relatively recent shared ancestry or significant gene flow among these three latter populations. Chinese, Pakistani and Nepalese populations were differentiated from one another and from the Middle Eastern and Mediterranean populations. These two latter populations were not highly differentiated from one another (Fig. S2; Table 2). The NW European population showed a strong differentiation from Mediterranean and Middle Eastern populations but was closer to the Chinese population (Fig. S2 and Fig. S3).

**Table 2:** Estimates of F_ST_ (upper diagonal) and its significance (lower diagonal) based on 20 microsetillite loci for 386 PST isolates representing worldwide geographically spaced populations.

	NW Europe	North America	South America	Mediterranean Region	Central Asia	South Africa	East Africa	Middle East	Nepal	Pakistan	China
**NW Europe**	-	**0.039**	**0.001**	0.420	0.380	0.498	0.500	0.380	0.370	0.410	0.390
**North America**	**0.100**	-	**0.046**	0.409	0.368	0.485	0.490	0.378	0.364	0.400	0.398
**South America**	**0.410**	**0.100**	-	0.435	0.396	0.514	0.511	0.393	0.379	0.416	0.405
**Mediterranean Region**	0.000	0.000	0.000	-	0.020	**0.109**	0.150	**0.009**	0.280	0.280	0.390
**Central Asia**	0.000	0.000	0.000	0.000	-	**0.044**	0.160	0.040	0.230	0.260	0.340
**South Africa**	0.000	0.000	0.000	**0.010**	**0.190**	-	**0.229**	0.160	0.298	0.314	0.419
**East Africa**	0.000	0.000	0.000	0.000	0.000	**0.600**	-	0.140	0.380	0.280	0.540
**Middle-East**	0.000	0.000	0.000	**0.020**	0.000	0.000	0.000	-	0.260	0.250	0.360
**Nepal**	0.000	0.000	0.000	0.000	0.000	0.000	0.000	0.000	-	0.220	0.210
**Pakistan**	0.000	0.000	0.000	0.000	0.000	0.000	0.000	0.000	0.000	-	0.450
**China**	0.000	0.000	0.000	0.000	0.000	0.000	0.000	0.000	0.000	0.000	-
**F**_ST_ **for aggressive strains**	0.420	0.408	0.434	**0.000**	**0.010**	**0.099**	0.150	**0.010**	0.270	0.270	0.390
**P-value for aggressive strains**	0.000	0.000	0.000	**0.860**	**0.070**	**0.040**	0.000	**0.020**	0.000	0.000	0.000

The lower two lines shows F_ST_ and its p-value for isolates representing the post-2000 emerged strains. Non-significant *F_ST_* values (>0.01) are shown in bold.

### Geographical patterns of genetic and genotypic variability

**Figure 3:**
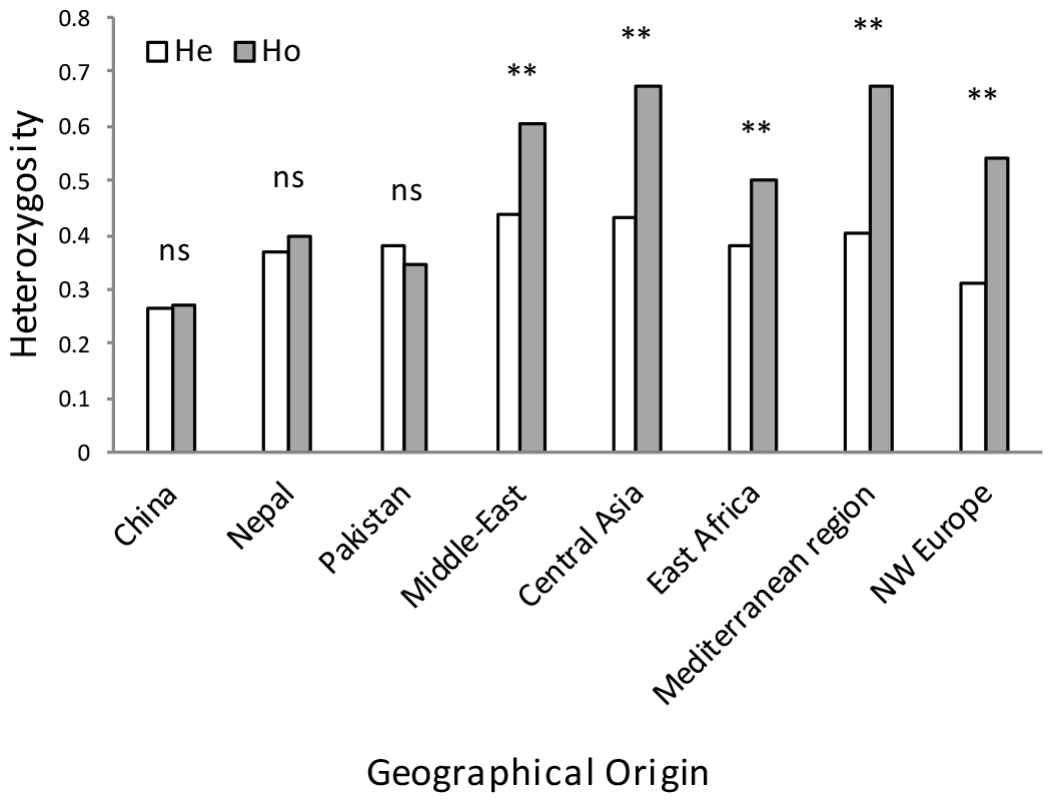
Expected (He) and observed (Ho) heterozygosity for clone-corrected data based on 20 polymorphic microsatellite loci for PST isolates sampled from diverse geographical regions.

**Figure 4:**
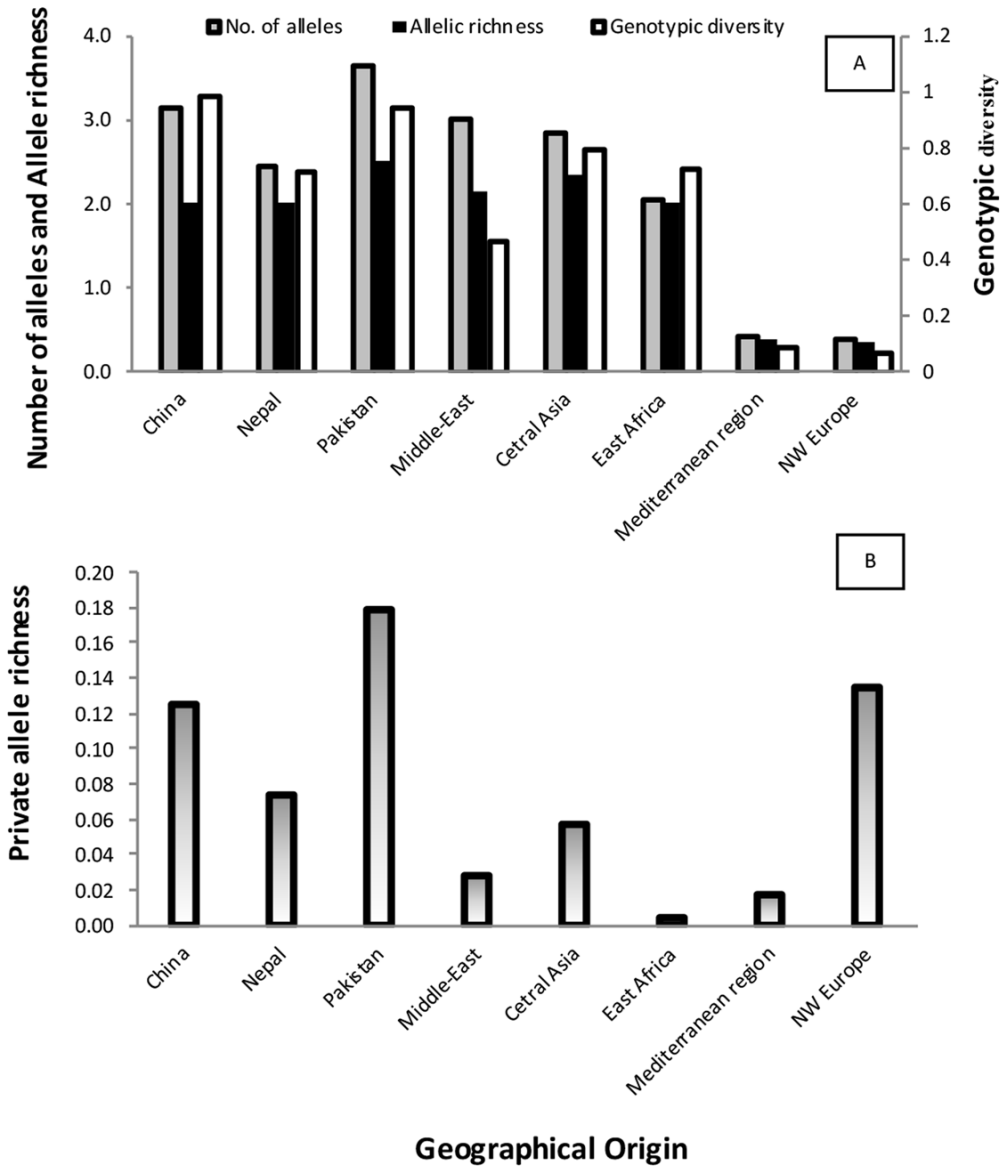
Diversity index, allele richness (A) and private allele richness (B) for PST populations from worldwide geographical regions.

Populations from NW Europe, North America, South America, Australia, South Africa, East Africa, the Middle East and the Mediterranean region displayed low genotypic diversity as well as an excess of heterozygosity compared to expectations under HWE, consistent with long-term clonality. Asia appeared as the zone of the highest diversity of the pathogen, with Himalayan (Nepalese and Pakistani) and near Himalayan (Chinese) populations not departing from HWE, suggesting the occurrence of recombination within the populations (Fig. 3), and showing higher variability in terms of genotypic diversity and allele richness (Fig. 4). Populations from Middle East and East Africa were more diverse than the European and Mediterranean populations, where the maximum clonal resampling was observed. Pakistan displayed the highest number of private alleles (Fig. 4). Isolates representing NW Europe also had high private allele richness, probably due to their strict clonality [19], [25] and isolation from other populations.

### Source of recently emerged populations

We detected only a few recent migrants, admixed and unassigned isolates in each geographical region in clustering analyses (Fig. 2 and Fig. S2). The existence of such genotypes in the Himalayan and neighboring regions may reflect back migrations or shared ancestral variation, as both phenomena give the same signal with clustering algorithms [60], [61]. Clear migration footprints were only found when focusing on recently colonized areas. Analyses confirmed NW Europe as the source of the North American and Australian populations, and the Mediterranean region and Central Asia appeared to be the source of the South African population (Fig. 2 and Fig. S2). Additionally, the South American isolates were assigned to NW European isolates and displayed very low diversity, revealing another incursion from NW Europe.

Isolates of the recently emerged aggressive strain PstS1, associated with the post-2000 epidemics in the USA and Australia, consisted of only a single multilocus genotype (MLG-99) resampled in other geographical regions as well. PstS1 was closely related to the other recently emerged aggressive strain reported in Asia, Africa and Europe, the PstS2, which consisted of different multilocus genotypes, including this MLG-99. Both PstS1 and PstS2 were assigned to the Middle Eastern-East Africa group, suggesting a source in Middle East-East Africa for these strains (Fig. 2 and Table 2). An older set of aggressive isolates frequently reported in Southern Europe [25], although with lesser number of virulences than PstS1 and PstS2 [24], were assigned to the Central Asian-Mediterranean genetic group (represented as PstS3 in Fig. 2).

### Ancestral relationship and migration patterns of populations

We used approximate Bayesian computation (ABC) analyses to infer on the ancestral relationship among populations. To limit the number and complexity of the scenarios to be compared, we used a sequential approach: we defined nested subsets of competing scenarios based on our understanding of the population structure in different regions, and analyzed these subsets sequentially. The origin of recently emerged populations was investigated using clustering and differentiation analyses and it has been presented in the previous section.

We started by analyzing the historical relationships among the three populations displaying recombining population structures and located in the region of highest diversity (i.e., Pakistan, Nepal and China). The four competing scenarios assumed three different population-trees, and admixture in the ancestry of the Nepalese population (Fig. S4) as this population appeared admixed in population subdivision analyses (Fig. S2 and Fig. S3). Leave-one-out cross-validation for model selection [52] confirmed that our methodology was able to distinguish between the four different scenarios (Table S5). The emergence of the Nepalese populations following admixture between Pakistani and Chinese populations appeared the most likely [Scenario 4a, posterior probability (PP): 0.9911; (Table S6)]. This scenario (4a) was then used as a backbone to design scenarios investigating the origin of other populations. Because a relatively recent origin of the Nepalese population was indicated by parameter estimates [Maximum Posterior Probability (MPP) estimate: 103 generations; 95% CI: 14–332; (Table S3)], this population was therefore dismissed as a possible source of other populations and not considered in subsequent analyses.

**Figure 5:**
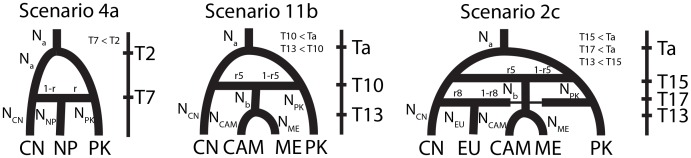
Ancestral relationship among worldwide PST populations as inferred from the analyses of Approximate Bayesian Computations.

The origin of Central Asian, Middle Eastern and Mediterranean population was investigated jointly, based on the relatedness of these populations in population structure analyses (Fig. 1, S2 and Table 2), and their geographical proximity from the centre of diversity (the Himalayan and neighboring areas). Central Asian and the Mediterranean populations were pooled, based on their genetic relatedness to limit the number of competing scenarios. Twelve scenarios were compared, assuming two different native populations (China and Pakistan), sequential or independent introductions and admixture (Fig. S5). Leave-one-out analyses indicated a good ability to distinguish between the twelve scenarios (Table S5). Scenario 11b, assuming that the Central Asian, Middle Eastern and Mediterranean populations split following an admixture event between Chinese and Pakistani populations appeared the most likely (PP = 0.717; Table S6; Fig. 5). The Pakistani population had a slightly higher contribution to the admixture event [1-r5 = 0.57, 95% Credibility interval (CI): 0.096–0.954] than the Chinese population, which might account for the assignment of simulated dataset under this scenario to scenario 10b (both populations originated from Pakistan) in leave-one-out analyses. We also considered the possibility that populations not represented in our dataset could have contributed to the genetic makeup of extant worldwide PST populations. All scenarios including a hypothetical un-sampled source population had lower posterior probabilities than the most supported scenario (11b) described above (scenarios set d; Fig. S7; Table S6). Leave-one-out analyses, however, indicated limited power to distinguish among the scenarios (Table S5).

The most likely scenario identified in analyses of Central Asian, Middle Eastern and Mediterranean populations was then used as a backbone to investigate the origin of the NW European population. Six scenarios were compared, assuming three different populations of origin (China, Pakistan and the Central-Asian-Middle-Eastern-Mediterranean lineage identified above) and admixture (Fig. S6). The scenario (2c) assuming that the NW European emerged following an admixture event between Chinese and Pakistani populations appeared the most likely (PP = 0.499; Table S6; Fig. 5). Parameter estimates for scenario 2c suggested a higher contribution of the Chinese population to the admixture event (1-r7 = 0.756, 95% CI: 0.187–0.987), which may account for the relatively high posterior probabilities for scenarios 1c and 4c [PP = 0.173 and PP = 0.270, respectively]. Inclusion of a hypothetical un-sampled source population revealed a slightly higher posterior probability of scenario 2c described above (PP = 0.303) than the posterior probability of scenario 4e (PP = 0.287) assuming that the NW European population resulted from an admixture event between the Chinese and un-sampled population (scenarios set e; Fig.S7; Table S6). Like scenario 2c, parameter estimates for scenario 4e suggested a higher contribution of the Chinese population to the admixture event (r8 = 0.797, 95%CI: 0.069–0.982).

## Discussion

We investigated the origin, migration routes and population structure of wheat yellow rust pathogen *Puccinia striiformis* f.sp. *tritici* (PST) using a comprehensive set of isolates from six continents and 20 highly informative SSR markers. We showed that despite the long-distance dispersal and the recent global spread of aggressive strains, the worldwide PST population has a clear genetic structure and is separated into six groups corresponding to main epidemic areas. The maintenance of a clear genetic structure despite substantial gene flow results from both strong clonality in many PST populations, and strong founder effects in recently emerged populations.

### Strong population subdivision despite long-distance dispersal

**Figure 6:**
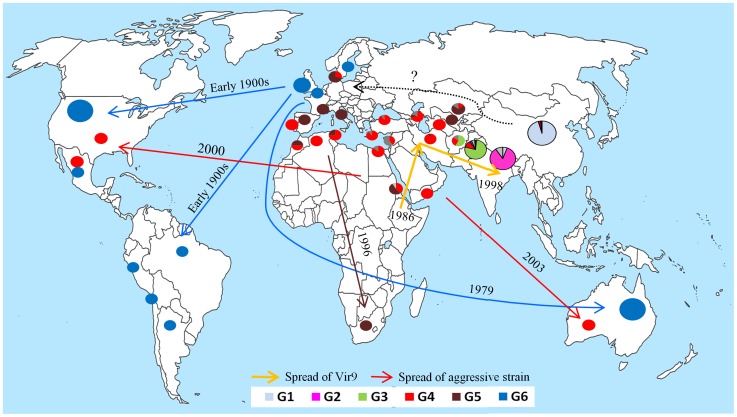
Origin and migration routes of recently emerged populations of wheat yellow rust pathogen identified or confirmed through population genetic analyses of a worldwide representative set of isolates. The year shows the first report of incidence based on present work and previous work (Aggressive strain = Hovmøller et al., 2011; South African population = Boshoff et al., 2002; Spread of Vir9 = Singh et al., 2004; incursion into Australia = Wellings and McIntosh, 1990).

We report the existence of a strong population subdivision within PST, with a clustering of isolates according to their geographical origin despite a capacity for long-distance migration [9], [11]. This pattern stands in stark contrast with the previous understanding of the worldwide population structure of PST, which considers the potential replacement of local populations by new invasions [5], [9]. On the basis of pathological survey that monitor the occurrence of strains with newly acquired virulences that defeat recently deployed *Yr* (resistance) genes, the population structure of PST was considered to be shaped by a continual replacement of pre-existing populations by the newly emerged and spread pathotypes, or aggressive strains. This process is well known as the boom and bust cycle [5], [10]. However, such surveys were designed to track the spread of a new, virulent race and, therefore, were potentially biased due to sampling only from varieties with the defeated resistance gene(s) in question but not from local landraces or other varieties. These observations lead to Asia (except China) being considered as a single epidemiological zone, with rapid and recurrent spread of new virulences over the whole zone, as in the case of virulence matching the *Yr9* resistance gene (Fig. 6 [5]) and the recent virulence matching the stem rust resistance gene *Sr31* [4]. Indeed, such geographic migrations are also documented in our study, but recently spread genotypes appear to coexist with and are dominated by older populations specific to the main geographic areas, suggesting that migrants do not replace local populations in recombinant Asian populations despite the capacity for recurrent and long-distance dispersal. In contrast, the invasion of new genotypes in clonal populations would result in a population sweep and would replace the original population. This was observed in the USA, where the post-2000 PST population is dominated by the pathotypes characteristic of the aggressive strain, PstS1, or its derivatives, shown above to have originated from the East African-Middle Eastern region. The outcome of new pathotype introductions may depend on the relative competitiveness of local populations in their region of origin, and the selective advantage of migrants (e.g. their virulence towards a new resistance gene widely deployed or an increased tolerance to prevalent abiotic stresses e.g., high temperatures).

### Regional differences in levels of recombination

PST has long been considered a strictly asexual pathogen on wheat due to the lack of knowledge about the existence of alternate host to complete sexual reproduction [16], [62]. Population genetic surveys that revealed clonal populations with very low diversity in USA [27], Europe [25] and Australia [15] were consistent with a hypothesis of asexual reproduction. Recently, populations with higher diversity were reported in the Middle East [31] and Pakistan [29], and a recombinant population structure was evidenced in China [28]. Herein, we identified a recombinant population structure and high diversity in Nepal and Pakistan and confirmed previous findings in China, suggesting the existence of possible sexual reproduction in PST populations from a broad area ranging from the Himalayan region to the Mongolian plateau. This possibility also recently gained indirect experimental support, with the demonstration of *Berberis spp.* as alternate host for PST under laboratory conditions [63] and a high ability for sexual reproduction (in the form of telial production) reported in the Asian populations of PST [32]. Although the role of *Berberis spp.* for the life cycle of PST under natural conditions remains to be further investigated, the presence of *Berberis spp.* in Pakistan, Nepal and China [64], [65], [66] is consistent with the existence of a sexual cycle of the pathogen in Asia.

### On the source of new incursions and emergences

Clustering and differentiation analyses allowed us to identify the source of new incursions and emergence (Fig. 2 and Fig. 6). The source of the Australian and North American populations was confirmed to be NW Europe, in accordance with previous findings, suggesting the migration of PST from NW Europe to Australia in 1979 [15] and probably earlier to North America [11], [67]. We also identified the NW European source of the South American PST population, which was reported earlier in the 20^th^ century with no inference on its source [16], [21]. This suggests that PST incursions into both North and South America originated from NW Europe, probably through human intervention. We also identified the Mediterranean-Central Asian population as the source of South African populations, first reported in 1996 [20], which might have resulted from wind dispersal or human intervention (Fig. 6).

Two closely related strains of PST, distinct from local populations, were recently reported in North America, Australia and Europe [6]. These strains were shown to be highly aggressive and adapted to high temperature [24]. One of the strains (PstS1) was responsible for PST epidemics in south-central USA, a region previously considered too warm for yellow rust epidemics [in 2000]; [ 22,68], and in Western Australia [in 2002; 69]. Another strain (PstS2), closely related to the first one, was reported in NW Europe with similar aggressiveness and strong differentiation from local PST populations. PstS2 was also shown to be present in the Mediterranean region and the Middle East-East Africa [6]. Our analyses revealed that PstS1 representative isolates had a single multilocus genotype (MLG-99), while PstS2 consisted of different, but closely related, MLGs. Assignment analyses revealed that both strains originated from the Middle East-East Africa, and such global patterns of dispersal would involve accidental spore transport linked with human activities. Thus, the incursion of PST into the Americas and the spread of aggressive strains are most probably the direct consequence of human-associated dispersal, as suggested for the initial introduction of PST in Australia [15]. These results suggests that PST may spread rapidly through winds or human activities, with former playing a greater role at regional scales [26], the latter could be involved in inter-continental spread.

### Origin of the worldwide PST populations

Transcaucasia had previously been suggested as the centre of origin for the pathogen, mainly based on its diversity of virulence and distribution of pathotypes [16], [17]. However, the diversity of virulence and the distribution of pathotypes are strongly influenced by the resistance in host populations, and this might lead to biased inferences of the location of the centre of origin of the pathogen. In our analyses, the representative isolates from Transcaucasia were less diverse, appeared to be clonal and did not exhibit strong divergence from the rest of the Middle-Eastern, Central Asian and Mediterranean populations. In contrast, the existence of high levels of diversity, private alleles, a recombinant population structure, the ability to produce sex-related structures [32] and the independent maintenance of PST populations in the Himalaya [66] suggest this latter region as a more plausible centre of origin for PST. The analysis of ancestral relationships among worldwide populations further confirmed the Himalayan populations to be the ancestral populations and the differentiation between Pakistan and China to be the most ancestral split (Fig. 5; Table S2, S3, S4, S5, S6). If one considers that the centre of origin of PST is in the Himalayan region, then PST would have adapted to wheat through host-range expansion or host shift and not followed a host-tracking co-evolution with early wheat domestication in the Fertile Crescent. This would add to the increasingly adopted view that infection of a novel host is a major route of disease emergence in fungal pathogens [70]. Additional sampling campaigns and full-genome re-sequencing of new isolates from wild and domesticated hosts should provide further insight into the history of PST and advance our understanding of the evolutionary response of pathogens to plant domestication and the development of agro-systems.

### Proposed scenario for historical migration routes of PST

Once adapted to wheat in its centre of origin in the Himalayas or the nearby regions, PST could have spread to the rest of the world while evolving independently in different parts of the Himalayan region, resulting in population subdivision within the native area [66]. PST could have spread northward from the Himalayas to the Mongolian plateau in China, where it maintained sexual reproduction and a high diversity in some parts, with an acquisition of virulences to the wheat varieties grown in China. Eastbound, ABC analyses indicated that relatively recent admixture event between Pakistani and Chinese populations resulted in the Nepali population. On the westward side of the Himalayan region, the three Middle-Eastern, Mediterranean and Central Asian populations could be the result of an admixture between Pakistan and China (Fig. 5) with relatively higher contribution of the Pakistani population to the admixture event according to ABC inferences. The Mediterranean, Middle Eastern and Central Asian populations were less differentiated, despite their distribution across a large geographical area. The off-season maintenance in some regions, its subsequent spread to other Middle-Eastern, Mediterranean and Central Asian regions and the lack of local off-season survival through volunteers or sexual reproduction in all regions could result in a source and sink relationship at the scale of this whole region, as previously suggested [31]. The most recent incursion from this Middle-Eastern, Mediterranean and Central Asian population was into South Africa, where PST was absent until 1996 [20].

The NW European population was shown in ABC analyses to be the result of admixture between China and Pakistan or between China an un-sampled source population with a higher contribution of the Chinese population to the admixture event. This is consistent with the lower differentiation between the NW European population and the Chinese population (Table 2), compared to the Pakistani or the Middle Eastern, Central Asian and Mediterranean populations. These results suggest that PST has spread from China to NW Europe, and this spread more likely occurred through some human intervention rather than an airborne incursion from the Middle Eastern, Central Asian and/or Mediterranean regions. This NW European population succeeded in terms of off-season survival on volunteers in coastal areas with mild winter and resulted in a reduced ability for sexual reproduction [32]. The clonal evolution within the NW European population resulted in a strong negative *F*_IS_ value, owing to a clonal divergence of the dikaryotic genomes [25], in line with what is expected according to the Meselson Effect [71]. From NW Europe, PST has subsequently been introduced to North and South America and even more recently to Australia (Fig. 6).

Our study suggests the Himalayan region as the likely centre of origin of PST, it confirms the previous migration hypotheses (invasion of USA and Australia from NW Europe and the source of aggressive strains), and it also provides an integrative scenario for the worldwide spread of PST including new findings regarding the origins of South American and European populations (Fig. 6). More extended surveys, particularly in northern parts of Asia and Eastern part of Himalayan region along with a multigene phylogeny approach based on several gene sequences will provide a more detailed resolution in the analysis of the colonization history of the pathogen and its population structure in Asia, taking advantage of the historical/epidemiological records of PST emergences.

### Conclusions

The existence of a high genotypic diversity, a high ability for sexual reproduction as well as the independent maintenance of strongly differentiated populations in the Himalayan region suggests this region as the putative centre of origin of PST. Differences in the levels of diversity and mode of reproduction among geographically distant populations are particularly relevant in the context of risk-assessment for disease emergence: Asian populations (China, Nepal and Pakistan) with a high level of recombination, diversity and ability for sexual reproduction could serve as possible sources for the emergence of new, virulent and aggressive strains. The maintenance of populations specific to geographical regions in Asia suggests a survival of local populations in these regions, potentially through sexual reproduction. For integrated disease management, it would be important to quantify the relative contribution of sexual vs. asexual reproduction to the diversity in different world populations and identify sexual host(s) or clonal over-summering/-wintering pathways. Finally, this study emphasizes the potential role of human travel and commerce as a major driver in the emergence of PST. The intensification of business and tourism activities in regions known as major sources of pathogen diversity should be considered in the context of risks associated with the emergence and worldwide spread of plant disease.

## Supporting Information

Figure S1 Box-plot of number of PST genotypes detected as a function of the number of loci re-sampled 1000 times within the 20 microsatellite markers (from 0–20) using GENCLONE software. The box represents the average, minimum and maximum numbers of MLGs detected when re-sampling on loci.
(DOC)

Figure S2 Assignment of PST isolates from worldwide geographical regions to genetic groups, using the STRUCTURE software and different K-values (genetic groups). The chart represents the consensus assignment obtained by analysis of result from 20 runs of the STRUCTURE analysis with the CLUMPP software. Each color represents a different genetic group.
(DOC)

Figure S3 Microsatellite distance-based Neighbor-Joining tree for PST isolates from worldwide geographically spaced populations. Agr = Aggressive strain, CA = Central Asia, Ch = China, Erit = East Africa, MdlEst = Middle East, Med = Mediterranean region, Nep = Nepal, NWEu = NW European group, Pk = Pakistan and SA = South Africa.
(DOC)

Figure S4 Scenarios regarding the evolutionary relationship among the three recombinant populations from the centre of diversity of *Puccinia striiformis* f.sp. *tritici*.
(DOC)

Figure S5 Scenarios regarding the evolutionary relationship between the two ancestral populations of Pakistan and China with the Middle Eastern and Central Asian-Mediterranean populations.
(DOC)

Figure S6 Scenarios regarding the evolutionary relationship between the two ancestral populations of Pakistan and China with Middle Eastern, Central Asian-Mediterranean and NW European populations.
(DOC)

Figure S7 Scenarios regarding the evolutionary relationship between the two ancestral populations of Pakistan and China with Middle Eastern, Central Asian-Mediterranean and NW European populations along with a ‘ghost’ population.
(DOC)

Table S1 Gene diversity for 20 SSR loci in PST populations of diverse geographical origin.
(XLS)

Table S2 Prior distributions of model parameters.
(XLS)

Table S3 Demographic and mutation parameter estimated using Approximate Bayesian Computation. Only parameter esimtates for the scenarios having highest posterior probabilities are shown. Demographic parameters are introduced in corresponding Figures. Composite parameters scaled by the mutation rate are also shown. The mutation parameters are μ (mean mutation rate) and p (mean value of the geometric distribution parameter that governs the number of repeated motifs that increase or decrease the length of the locus during mutation events).
(XLS)

Table S4 Model checking for demographic scenarios analysed using Approximate Bayesian Computation.
(XLS)

Table S5 Confidence in model choice for the demographic scenarios compared using approximate Bayesian computations. Confidence in model choice was assessed using a leave-one-out method. For each scenario 100 of the 500,000 simulated datasets used for model selection were randomly drawn and treated as pseudo-observed datasets. Posterior probabilities of competing scenarios were evaluated for each pseudo-observed dataset, using all remaining simulated datasets. Confidence in model choice was then estimated as the proportions of simulated data assigned to each scenario.
(XLS)

Table S6 Posterior probabilities (PP) of the different sets of scenarios compared using Approximate Bayesian Computation.
(XLS)
