# Supplementary material for: Origin, Migration Routes and Worldwide Population Genetic Structure of the Wheat Yellow Rust Pathogen Puccinia striiformis f.sp. tritici
Source: PLoS Pathog. 2014 Jan 23;10(1):e1003903. doi: 10.1371/journal.ppat.1003903 (PMC3900651; doi:10.1371/journal.ppat.1003903)
Supplement: Figure S1 — Box-plot of number of PST genotypes detected as a function of the number of loci re-sampled 1000 times within the 20 microsatellite markers (from 0–20) using GENCLONE software. The box represents the average, minimum and maximum numbers of MLGs detected when re-sampling on loci. (DOC) [file ppat.1003903.s001.doc]

Figure S1. Box-plot of number of PST genotypes detected as a function of the number of loci re-sampled 1000 times within the 20 microsatellite markers (from 0 -20) using GENCLONE software. The box represents the average, minimum and maximum numbers of MLGs detected when re-sampling on loci.
