# Supplementary material for: Origin, Migration Routes and Worldwide Population Genetic Structure of the Wheat Yellow Rust Pathogen Puccinia striiformis f.sp. tritici
Source: PLoS Pathog. 2014 Jan 23;10(1):e1003903. doi: 10.1371/journal.ppat.1003903 (PMC3900651; doi:10.1371/journal.ppat.1003903)
Supplement: Figure S2 — Assignment of PST isolates from worldwide geographical regions to genetic groups, using the STRUCTURE software and different K-values (genetic groups). The chart represents the consensus assignment obtained by analysis of result from 20 runs of the STRUCTURE analysis with the CLUMPP software. Each color represents a different genetic group. (DOC) [file ppat.1003903.s002.doc]

Figure S2. Assignment of PST isolates from worldwide geographical regions to genetic groups, using the STRUCTURE software and different K-values (genetic groups). The chart represents the consensus assignment obtained by analysis of result from 20 runs of the STRUCTURE analysis with the CLUMPP software. Each color represents a different genetic group.
