# Supplementary material for: Origin, Migration Routes and Worldwide Population Genetic Structure of the Wheat Yellow Rust Pathogen Puccinia striiformis f.sp. tritici
Source: PLoS Pathog. 2014 Jan 23;10(1):e1003903. doi: 10.1371/journal.ppat.1003903 (PMC3900651; doi:10.1371/journal.ppat.1003903)
Supplement: Figure S3 — Microsatellite distance-based Neighbor-Joining tree for PST isolates from worldwide geographically spaced populations. Agr = Aggressive strain, CA = Central Asia, Ch = China, Erit = East Africa, MdlEst = Middle East, Med = Mediterranean region, Nep = Nepal, NWEu = NW European group, Pk = Pakistan and SA = South Africa. (DOC) [file ppat.1003903.s003.doc]

Figure S3. Microsatellite distance-based Neighbor-Joining tree for PST isolates from worldwide geographically spaced populations. Agr = Aggressive strain, CA = Central Asia, Ch = China, Erit = East Africa, MdlEst = Middle East, Med = Mediterranean region, Nep = Nepal, NWEu = NW European group, Pk = Pakistan and SA = South Africa.
