# Supplementary material for: Origin, Migration Routes and Worldwide Population Genetic Structure of the Wheat Yellow Rust Pathogen Puccinia striiformis f.sp. tritici
Source: PLoS Pathog. 2014 Jan 23;10(1):e1003903. doi: 10.1371/journal.ppat.1003903 (PMC3900651; doi:10.1371/journal.ppat.1003903)
Supplement: Figure S4 — Scenarios regarding the evolutionary relationship among the three recombinant populations from the centre of diversity of Puccinia striiformis f.sp. tritici. (DOC) [file ppat.1003903.s004.doc]

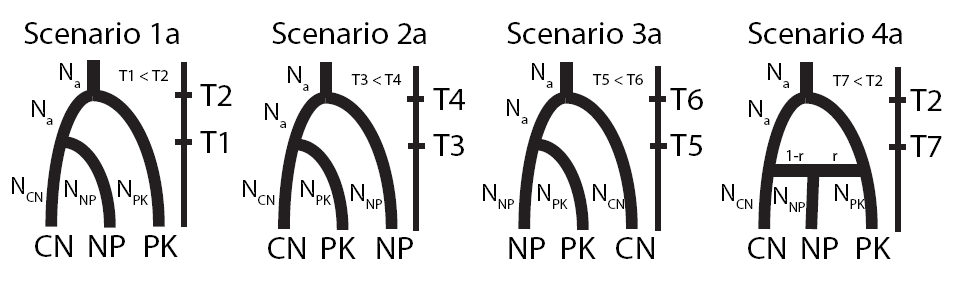


Figure S4. Scenarios regarding the evolutionary relationship among the three recombinant populations from the centre of diversity of *Puccinia striiformis* f.sp. *tritici*.
