# Supplementary material for: Origin, Migration Routes and Worldwide Population Genetic Structure of the Wheat Yellow Rust Pathogen Puccinia striiformis f.sp. tritici
Source: PLoS Pathog. 2014 Jan 23;10(1):e1003903. doi: 10.1371/journal.ppat.1003903 (PMC3900651; doi:10.1371/journal.ppat.1003903)
Supplement: Figure S5 — Scenarios regarding the evolutionary relationship between the two ancestral populations of Pakistan and China with the Middle Eastern and Central Asian-Mediterranean populations. (DOC) [file ppat.1003903.s005.doc]

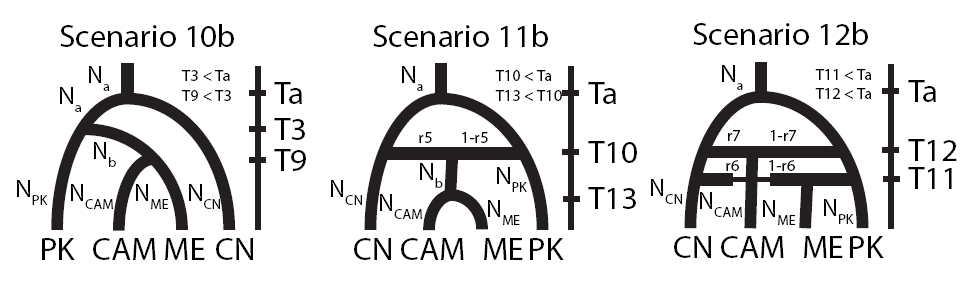

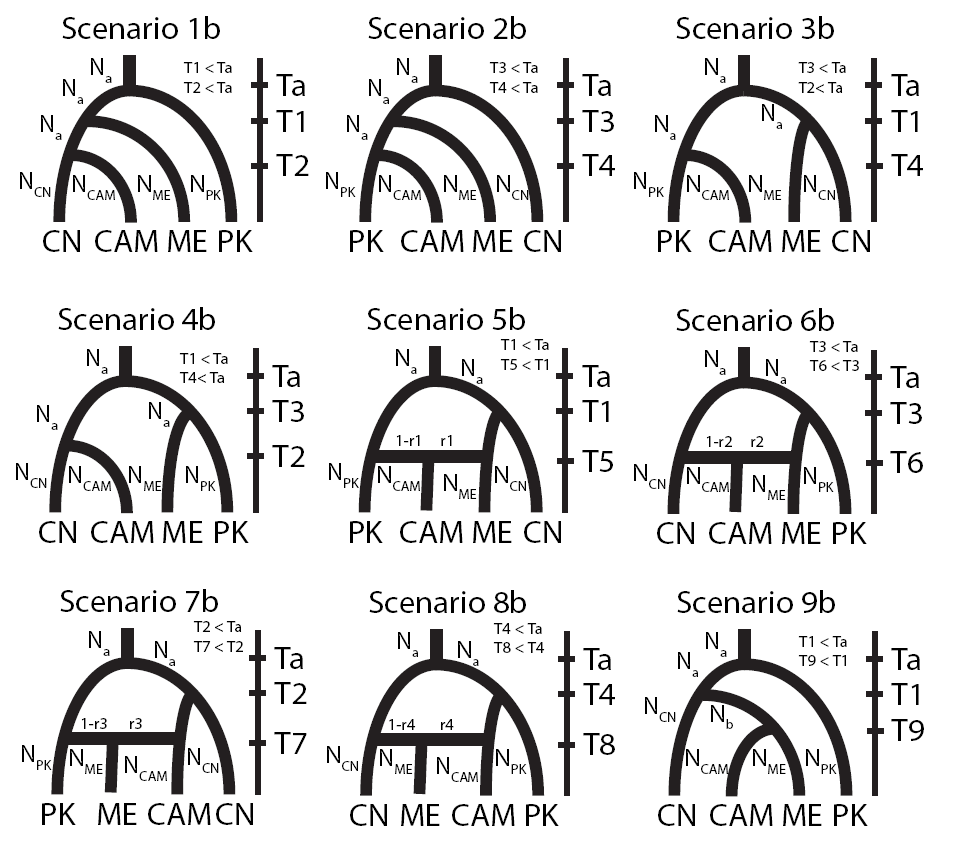


Figure S5. Scenarios regarding the evolutionary relationship between the two ancestral populations of Pakistan and China with the Middle Eastern and Central Asian-Mediterranean populations.
