# Supplementary material for: Origin, Migration Routes and Worldwide Population Genetic Structure of the Wheat Yellow Rust Pathogen Puccinia striiformis f.sp. tritici
Source: PLoS Pathog. 2014 Jan 23;10(1):e1003903. doi: 10.1371/journal.ppat.1003903 (PMC3900651; doi:10.1371/journal.ppat.1003903)
Supplement: Figure S6 — Scenarios regarding the evolutionary relationship between the two ancestral populations of Pakistan and China with Middle Eastern, Central Asian-Mediterranean and NW European populations. (DOC) [file ppat.1003903.s006.doc]

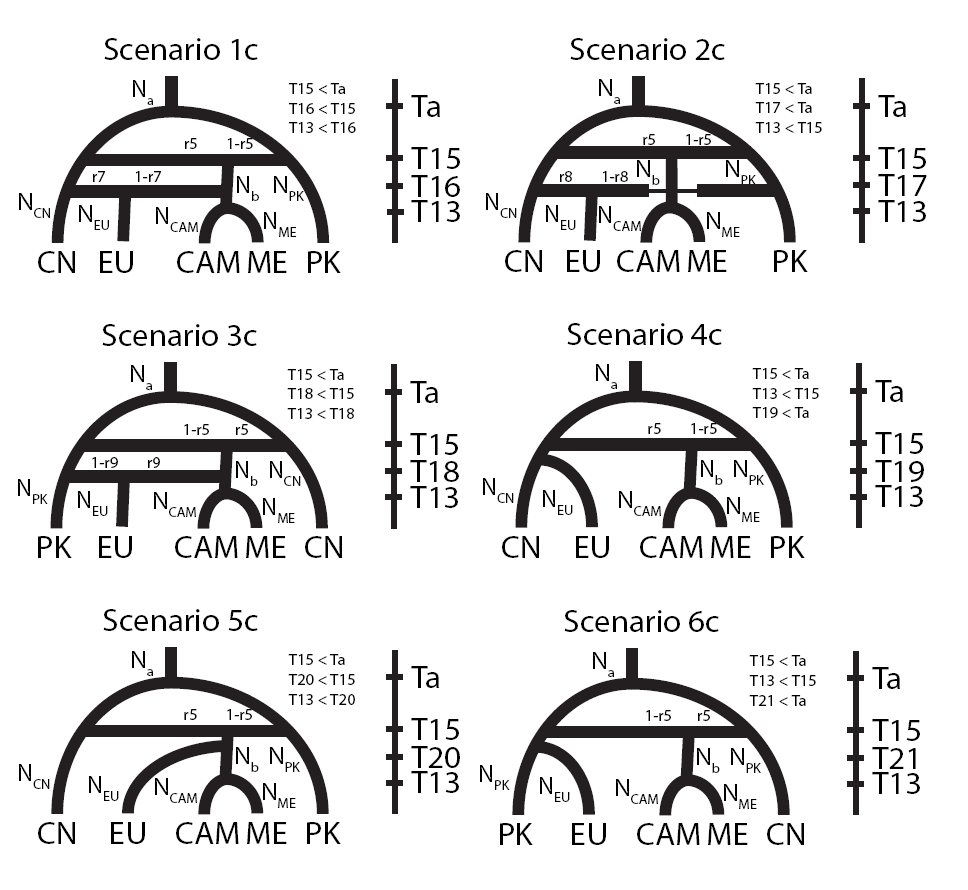


Figure S6. Scenarios regarding the evolutionary relationship between the two ancestral populations of Pakistan and China with Middle Eastern, Central Asian-Mediterranean and NW European populations.
